# Editorial: Cellular and molecular mechanisms of lung regeneration, repair, and fibrosis, volume II

**DOI:** 10.3389/fcell.2025.1653034

**Published:** 2025-08-12

**Authors:** Yong Qiu, Xiao Xiao Tang, Shigeyuki Shichino, Prem Prakash Kushwaha, Yan Y. Sanders, Remo Castro Russo, Chunheng Mo

**Affiliations:** ^1^ Key Laboratory of Birth Defects and Related Diseases of Women and Children of MOE, State Key Laboratory of Biotherapy, West China Second University Hospital, Sichuan University, Chengdu, China; ^2^ Department of Anesthesiology, Zigong First People's Hospital, Zigong Academy of Medical Sciences, Zigong, Sichuan, China; ^3^ State Key Laboratory of Respiratory Disease, National Clinical Research Center for Respiratory Disease, National Center for Respiratory Medicine, Guangzhou Institute of Respiratory Health, The First Affiliated Hospital of Guangzhou Medical University, Guangzhou, China; ^4^ Guangzhou Laboratory, Guangzhou, China; ^5^ Division of Molecular Regulation of Inflammatory and Immune Diseases, Research Institute of Biomedical Sciences, Tokyo University of Science, Chiba, Japan; ^6^ School of Medicine, Case Western Reserve University, Cleveland, United States; ^7^ Department of Biomedical and Translational Sciences, Eastern Virginia Medical School, Old Dominion University Norfolk, Norfolk, VA, United States; ^8^ Laboratory of Pulmonary Immunology and Mechanics, Department of Physiology and Biophysics, Institute of Biological Sciences, Federal University of Minas Gerais (UFMG), Belo Horizonte, Brazil

**Keywords:** lung fibrosis, lung regeneration, bronchopulmonary dysplasia, asthma, chronic obstructive pulmonary disease

Organ fibrosis represents a substantial a major contributor to global disease burden, implicated in over 30% of cases leading to disability-associated and fatal conditions. Among these conditions, idiopathic pulmonary fibrosis (IPF) stands as a progressive and frequently fatal interstitial lung disease characterized by aberrant extracellular matrix deposition and parenchymal scarring. The prognosis for IPF patients is poor, with a median survival time of just 3–5 years (Bhattacharya and Ramachandran, 2023; Lederer and Martinez, 2018, Ma et al., 2024, Wei et al., 2024). Current FDA-approved therapeutics, Pirfenidone and Nintedanib, demonstrate capacity to attenuate functional decline in pulmonary fibrosis but remain disease-modifying rather than curative. Consequently, elucidating the underlying cellular and molecular mechanisms while developing more effective treatments constitutes an imperative research priority (Bonella et al., 2023, Podolanczuk et al., 2023, Zhang et al., 2023, Zhou et al., 2024).

**FIGURE 1:**
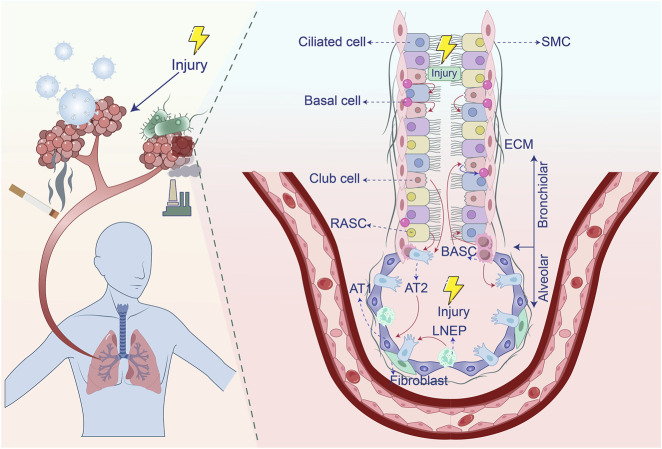
Stem cell populations involved in lung regeneration and repair after injury. After airway damage, basal cells have the capacity to differentiate into both club cells and ciliated cells. Club cells, in turn, can give rise to ciliated cells and are capable of dedifferentiating into basal cells when basal cells are lost. BASCs contribute to the regeneration of both club cells and ciliated cells. In response to alveolar injury, Club cells and BASCs play a key role in replenishing AT2 cells. LNEPs are able to differentiate into AT2 cells, while RASCs primarily give rise to AT2 cells. Additionally, AT2 cells have the ability to differentiate into AT1 cells. Extracellular matrix, ECM; Type 1 alveolar epithelial cells, AT1; Type 2 alveolar epithelial cells, AT2; Alveolar macrophages, AM; Smooth muscle cell, SMC; Lineage-negative epithelial progenitors, LNEPs; Bronchioalveolar stem cells, BASCs; Respiratory airway secretory cells, RASCs.

Impaired pulmonary regeneration and repair mechanisms represent critical determinants in the pathogenesis of lung injury-induced fibrosis. As the primary interface for gas exchange, lungs process >10,000 L of air daily to facilitate oxygen delivery and carbon dioxide clearance. This direct environmental exposure predisposes pulmonary tissue to damage from diverse insults including airborne pollutants, tobacco smoke, chemical agents, and viral or bacterial pathogens (Mo et al., 2024, Moss et al., 2022, Neupane et al., 2020). Post-injury, the lungs activate intrinsic regenerative programs by recruiting multiple stem cell populations—such as type 2 alveolar epithelial (AT2) cells, basal cells, club cells, lineage-negative epithelial progenitors (LNEPs), bronchioalveolar stem cells (BASCs), and respiratory airway secretory cells (RASCs). Efficient execution of these repair cascades is essential for restoring pulmonary homeostasis. However, persistent injury or dysregulated healing responses can compromise regenerative capacity, promoting pathological extracellular matrix deposition that manifests as fibrotic scarring and progressive functional impairment (Basil et al., 2020, Chen et al., 2024, Jones et al., 2024, Liu et al., 2024, Niethamer et al., 2025, Wei et al., 2025) (Figure 1). Elucidating the cellular and molecular basis of lung regeneration and repair is therefore fundamental for deciphering fibrotic pathogenesis and designing targeted therapies.

Current understanding of the cellular and molecular mechanisms governing lung regeneration, repair, and fibrosis remains incomplete. This Research Topic addresses this pressing knowledge gap through two original research article and two comprehensive reviews, collectively advancing mechanistic insights into these interconnected processes. The presented findings elucidate novel aspects of pulmonary regenerative biology and fibrotic pathogenesis while proposing translational diagnostic frameworks and targeted therapeutic strategies for pulmonary fibrosis and related interstitial lung diseases.

Early intervention is critical for effective IPF management. Improving outcomes necessitates identifying precise biomarkers and targets, particularly in early disease stages, to enable timely therapeutic strategies (Karampitsakos et al., 2023, Oldham et al., 2024). Huang et al. synthesized molecular and clinical evidence highlighting SIRT3, SIRT6, and SIRT7 as pivotal regulators in pulmonary fibrosis pathogenesis. These sirtuins exhibit significantly reduced expression in IPF patient-derived fibroblasts, with SIRT7 demonstrating the most pronounced deficiency. SIRT3/6/7 deficiencies disrupt mitochondrial homeostasis, promote oxidative stress, and drive fibroblast-to-myofibroblast transition (FMT) and epithelial-mesenchymal transition (EMT) via dysregulation of TGF-β/Smad, NF-κB, and cGAS-STING pathways. A “sirtuin activity score,” reflecting expression and functional status of SIRT3/6/7 and their downstream targets (e.g., SOD2, OGG1, Smad2/3), correlates significantly with clinical fibrosis severity and disease progression. Pharmacological activation of SIRT3 (e.g., by honokiol, resveratrol) or SIRT6 (e.g., by IMU-856) attenuates mitochondrial damage, inflammation, and fibrogenesis in preclinical models, demonstrating therapeutic potential. In addition, Clinical trials (e.g., NCT05417061 for SIRT6 activators) are evaluating sirtuin-targeted interventions, supported by biomarker analyses showing SIRT3 cytoplasmic levels predict disease outcomes in related conditions. These findings establish SIRT3/6/7 as multifunctional biomarkers and therapeutic targets, offering new diagnostic avenues and mechanistic insights into IPF pathogenesis, while paving the way for sirtuin-modulating therapies to alter disease trajectories.

Bronchopulmonary dysplasia (BPD) represents a developmental disorder of prematurity characterized by impaired alveolarization and pulmonary microvascular dysgenesis, stemming from disrupted lung development and aberrant repair mechanisms. Successful lung regeneration and structural maturation require coordinated signaling within the alveolar niche, involving precise epithelial-endothelial-mesenchymal crosstalk (Gilfillan et al., 2021, Sucre et al., 2018). However, dysregulation of hepatocyte growth factor (HGF)-mediated signaling in the epithelial-endothelial-mesenchymal network disrupts these critical interactions, exacerbating alveolar simplification and fibrosis. Sang and Qiao investigated the role of HGF/c-Met signaling in BPD pathogenesis. They analyzed hyperoxia-induced BPD models and observed that HGF deficiency diminished pulmonary vascular density and promoted epithelial-mesenchymal transition (EMT). Conversely, recombinant HGF (rhHGF) administration activated downstream PI3K/Akt and ERK pathways, stimulating angiogenesis and suppressing TGFβ-driven EMT. Furthermore, HGF synergized with VEGF to stabilize nascent vasculature and countered ECM stiffness by upregulating MMP-9. These findings demonstrate that HGF insufficiency disrupts vascular-epithelial crosstalk, impeding lung repair in BPD. Critically, HGF augmentation promotes alveolar maturation through dual mechanisms: pro-angiogenic activation and anti-fibrotic pathway modulation.

Circular RNAs (circRNAs) represent emerging candidates as diagnostic biomarkers and therapeutic targets in asthma pathogenesis. Non-coding RNAs, including miRNAs and circRNAs, play crucial roles in regulating airway inflammation and remodeling. Alongside miRNA-mediated gene silencing, the competing endogenous RNA (ceRNA) mechanism has emerged as a key regulatory pathway in asthma pathogenesis (Jia et al., 2024, Liu et al., 2023). Liu et al. investigated the role of circ-0001454 in asthma. Circ-0001454 was characterized by its cytoplasmic localization, ability to sponge miRNAs, and involvement in modulating oxidative stress and mitochondrial function. They identified a negative correlation between circ-0001454 and miR-770-5p, demonstrating that circ-0001454 acts as a molecular sponge to sequester miR-770-5p, thereby alleviating its suppression of the target gene cbl-b. They also established that the circ-0001454/miR-770-5p/cbl-b axis regulates downstream signaling proteins (EGFR, AKT1, MAPK1), reducing inflammation, oxidative stress, apoptosis, and mitochondrial dysfunction in bronchial epithelial cells and murine models. *In vivo*, circ-0001454 overexpression significantly attenuated airway hyperresponsiveness, inflammatory cytokine levels (IL-4, IL-5, IL-13), mucus hypersecretion, collagen deposition, and α-SMA expression. These findings establish circ-0001454 as a pleiotropic regulator of asthmatic pathophysiology while highlighting its translational potential as both a biomarker and RNA-based therapeutic target for mitigating airway inflammation and remodeling.

In addition to asthma and lung fibrosis, chronic obstructive pulmonary disease (COPD), characterized by persistent airflow limitation and chronic airway inflammation often linked to smoking, represents a major cause of global lung function impairment. Transcriptomic analysis of differentiating airway epithelial cells provides a powerful tool for identifying key regulators of COPD-associated epithelial remodeling (Lahmar et al., 2022, Petit et al., 2023). Laure et al. employed this approach combined with Hedgehog pathway inhibition to investigate altered epithelial repair mechanisms in COPD. They highlighted POU5F1 (OCT3/4) as a critical factor bridging Hedgehog signaling and epithelial plasticity. Critically, the alteration of POU5F1 was validated in primary COPD airway epithelial cells and lung tissues. Although deeper exploration is needed, this discovery uncovers essential clues linking Hedgehog signaling dysregulation to the defective epithelial remodeling characteristic of COPD pathogenesis. Thus, POU5F1-mediated signaling contributes to impaired regenerative capacity. Therefore, these findings establish Hedgehog-POU5F1 axis dysregulation as a fundamental mechanism driving pathological remodeling in COPD, revealing actionable targets for therapeutic intervention.

Collectively, the research presented in this Research Topic elucidates fundamental cellular and molecular mechanisms governing lung regeneration, aberrant repair, and fibrotic pathogenesis. Furthermore, these contributions significantly advance therapeutic development for pulmonary fibrosis and enhance diagnostic approaches for affected patients. These integrated discoveries establish new conceptual frameworks for intercepting fibrotic progression and restoring pulmonary homeostasis.
